# Association between Serum Soluble Klotho Levels and Mortality in Chronic Hemodialysis Patients

**DOI:** 10.1155/2015/406269

**Published:** 2015-10-28

**Authors:** Naoko Otani-Takei, Takahiro Masuda, Tetsu Akimoto, Sumiko Honma, Yuko Watanabe, Kazuhiro Shiizaki, Takuya Miki, Eiji Kusano, Yasushi Asano, Makoto Kuro-o, Daisuke Nagata

**Affiliations:** ^1^Division of Nephrology, Department of Internal Medicine, Jichi Medical University, Shimotsuke, Tochigi 329-0498, Japan; ^2^Department of Nephrology, Japanese Red Cross Koga Hospital, Koga, Ibaraki, Japan; ^3^Division of Anti-Aging Medicine, Center for Molecular Medicine, Jichi Medical University, Shimotsuke, Tochigi 329-0498, Japan

## Abstract

Klotho is a single-pass transmembrane protein predominantly expressed in the kidney. The extracellular domain of Klotho is subject to ectodomain shedding and is released into the circulation as a soluble form. Soluble Klotho is also generated from alternative splicing of the *Klotho* gene. In mice, defects in Klotho expression lead to complex phenotypes resembling those observed in dialysis patients. However, the relationship between the level of serum soluble Klotho and overall survival in hemodialysis patients, who exhibit a state of Klotho deficiency, remains to be delineated. Here we prospectively followed a cohort of 63 patients with a mean duration of chronic hemodialysis of 6.7 ± 5.4 years for a median of 65 months. Serum soluble Klotho was detectable in all patients (median 371 pg/mL, interquartile range 309–449). Patients with serum soluble Klotho levels below the lower quartile (<309 pg/mL) had significantly higher cardiovascular and all-cause mortality rates. Furthermore, the higher all-cause mortality persisted even after adjustment for confounders (hazard ratio 4.14, confidence interval 1.29–13.48). We conclude that there may be a threshold for the serum soluble Klotho level associated with a higher risk of mortality.

## 1. Introduction

Klotho is a single-pass transmembrane protein with a long extracellular domain and short cytoplasmic tail that appears to modulate aging [1, 2]. Despite the specific predominant expression of Klotho in the kidney, parathyroid gland, and choroid plexus of the brain, the extracellular domain of Klotho may be cleaved and released into the blood, cerebrospinal fluid, and urine in a soluble form [3–7], presumably allowing Klotho to participate in processes of pleiotropic pathophysiological regulation [1]. Overexpression of the* Klotho* gene extends longevity, while defective Klotho proteins are associated with premature death [2, 8]. Variations in the deoxyribonucleic acid sequences of* Klotho* have been shown to be associated with various pathologies, including osteoporosis, stroke, and coronary artery disease [9–11]. Furthermore, these polymorphisms are linked to poor survival in some subsets of patients with advanced chronic kidney disease (CKD) [12, 13]. Serum soluble Klotho (sKlotho) also possibly plays a role in determining the risk of cardiovascular disease or mortality in some populations and animal models [14–18]. However, information regarding the impact of serum sKlotho levels on the clinical characteristics of CKD patients is poor. In the present study, we therefore investigated the predictive significance of varied serum sKlotho levels in chronic hemodialysis (HD) patients in terms of survival and cardiovascular events.

## 2. Materials and Methods

### 2.1. Study Population

The study population consisted of male and female chronic HD patients treated at Japanese Red Cross Koga Hospital, Ibaraki, Japan, recruited for a prospective single-center study. The rationale, design, and data collection procedures of the study have been described elsewhere [19]. Exclusion criteria for all participants were as follows: active malignancy, pulmonary disease, peritoneal dialysis, death within three months after study entry, and failure to cooperate with the study or provide consent to participate. A total of 63 subjects with archived serum samples available at the point of enrollment were included in the current study. This prospective study was conducted in accordance with the Declaration of Helsinki. The research protocol was approved by the Medical Ethics Committee of Japanese Red Cross Koga Hospital, and all patients provided written informed consent.

### 2.2. Data Collection

Demographic and medical data, including age, gender, smoking history, and comorbid conditions, were obtained from the subjects' medical records in addition to standardized interviews. The body mass index (BMI) was calculated from the weight and height measurements as the weight (kg) divided by the square of the height (m^2^). Blood samples were obtained before HD on the first dialysis day of the week. Patients had been in the supine position for at least 10 minutes before blood collection. Aliquots of serum were obtained immediately at study entry and stored at −80°C until further use. Laboratory data included the levels of hemoglobin (Hb), serum albumin (sAlb), total cholesterol, serum calcium (sCa), serum phosphorus (sPi), intact parathyroid hormone (iPTH), and 1,25-dihydroxyvitamin D [1,25(OH)_2_D]. The normalized protein catabolism rate (nPCR) and the *Kt*/*V* urea index, which integrates the efficiency of solute removal as urea clearance (*K*), treatment duration (*T*), and the patient's size as urea distribution volume (*V*), were determined using previously described formulas [19, 20]. Blood pressure was measured before HD using a calibrated digital scale. The serum levels of sKlotho were determined according to a solid-phase sandwich enzyme-linked immunosorbent assay (ELISA) kit (Immuno-Biological Laboratories, Gunma, Japan), as described previously [5–7, 14–16]. The serum intact fibroblast growth factor 23 (FGF23) levels were determined using a sandwich ELISA kit (Kainos Laboratories Inc., Tokyo, Japan) according to the manufacturer's instructions [5]. Because the effective measurement range of this assay was between 3 and 800 pg/mL, serum samples with high FGF23 (>800 pg/mL) were diluted appropriately with the assay buffer. The measurements of the serum iPTH and 1,25(OH)_2_D levels were performed by a commercially based clinical diagnostic testing service (SRL, Inc., Tokyo, Japan).

### 2.3. Outcome Measurements

The primary outcomes were all-cause mortality and the first episode of fatal or nonfatal cardiovascular events, which were defined as angina or myocardial infarction (according to the Third Universal Definition of Myocardial Infarction) [21]; congestive heart failure requiring hospitalization (admission for a clinical syndrome involving symptoms in conjunction with clinical or radiologic signs of heart failure) [22]; transient ischemic attacks (a transient episode of neurologic dysfunction caused by focal brain, spinal cord, or retinal ischemia, without acute infarction) [23]; stroke (rapidly developing clinical symptoms or signs of focal [or at times global] disturbance of cerebral function lasting 24 hours [unless interrupted by surgery] or leading to death, with no apparent cause other than vascular origin) [24]; peripheral vascular disease, major arterial/venous thrombotic episode, and sudden death. For patients with multiple cardiovascular events, the time to the first episode was taken for survival analysis. Cardiovascular death was defined as death from any cardiovascular event. Cardiovascular events and cause of death were assessed by the attending physicians who were unaware of serum Klotho and FGF23 levels. The information in the patients' medical charts was also included in the analysis. Prognostic information for patients transferred to other dialysis clinics during the observation period was obtained from interviews with each new attending doctor. The patients' usual medications, including antihypertensive agents, such as angiotensin receptor blockers (ARBs), angiotensin-converting enzyme inhibitors (ACE-Is) and calcium channel blockers (CCBs), erythropoiesis-stimulating agents (ESAs), active vitamin D sterols, and phosphate binders, were continued and titrated as necessary during the observation period. Follow-up was completed for all patients.

### 2.4. Statistical Analyses

The data are expressed as either the number of participants or the percentage of the study population. The remaining data are expressed as the mean ± standard deviation (SD), or median and interquartile range (IR) for variables with a skewed distribution. The groups were compared using a one-way analysis of variance for normal distributions and the Kruskal-Wallis rank test for skewed distributions. The chi-square test was used to evaluate the proportional differences in categorical variables. Prior to conducting the survival analysis, the 25th, 50th, and 75th percentiles of serum sKlotho and serum FGF23 levels in the current study population were determined. The subjects were categorized as follows: low-Klotho group (low-KL), subjects with a serum sKlotho level under the 25th percentile; middle-Klotho group (middle-KL), subjects with a serum sKlotho level equal or over the 25th percentile and under the 75th percentile; high-Klotho group (high-KL), subjects with a serum sKlotho level of equal to or over the 75th percentile; low-FGF23 group (low-FGF23), subjects with a serum FGF23 level under the 25th percentile; middle-FGF23 group (middle-FGF23), subjects with a serum FGF23 level equal to or over the 25th percentile and under the 75th percentile; high-FGF23 group (high-FGF23), subjects with a serum FGF23 level of equal to or over the 75th percentile. We then assessed the impact of the serum sKlotho and FGF23 levels on the cumulative patient and cardiovascular event-free survival rates according to the Kaplan-Meier method combined with the log-rank test. Cox regression models were used to analyze the relationships between the primary outcomes and the serum sKlotho levels. Multivariate models were also applied to adjust for age, gender, diabetes mellitus, sPi, sAlb, and Hb, which are known to be associated with the risk of mortality in patients under chronic HD treatment [16]. The results are presented as the hazard ratio (HR) and 95% confidence interval (CI). *p* values of less than 0.05 were considered to be statistically significant. The statistical analyses were performed using the JMP software package version 9 (SAS Institute, Cary, NC), unless otherwise specified.

## 3. Results

### 3.1. Patient Characteristics

Soluble Klotho was detectable in the serum of all 63 chronic HD patients, with serum concentrations of sKlotho ranging from 167 to 720 pg/mL (median 371, IR 309–449). No significant difference in serum sKlotho levels was observed between males and females. The baseline clinical and demographic profiles of the subjects with the three varied degrees of serum sKlotho levels are summarized in Table 1. There were significant differences in the serum levels of FGF23 and the prevalence of use of ARBs or ACE-Is, whereas the other parameters were comparable among the three groups. The underlying causes of CKD included chronic glomerulonephritis in 28 patients (44%), diabetic nephropathy in 21 patients (33%), hypertensive nephrosclerosis in three patients (5%), and other causes in 11 patients (18%).

### 3.2. Cardiovascular Events and Mortality

**Figure 1:**
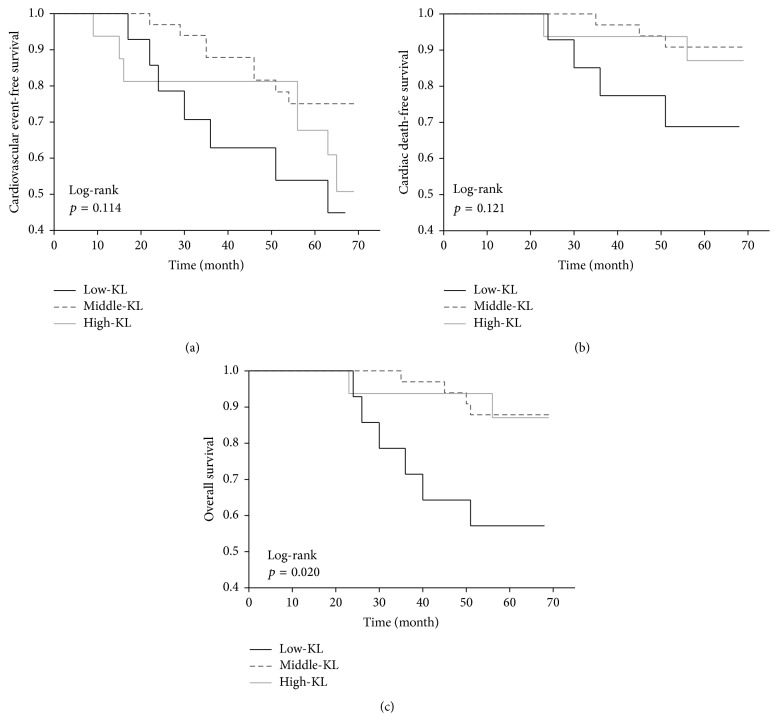
Kaplan-Meier plots of cardiovascular event-free survival (a), cardiovascular mortality (b), and cumulative survival (c). The patients were categorized into low-KL (<309 pg/mL), middle-KL (309 to <449 pg/mL), and high-KL (≥449 pg/mL) groups.

**Figure 2:**
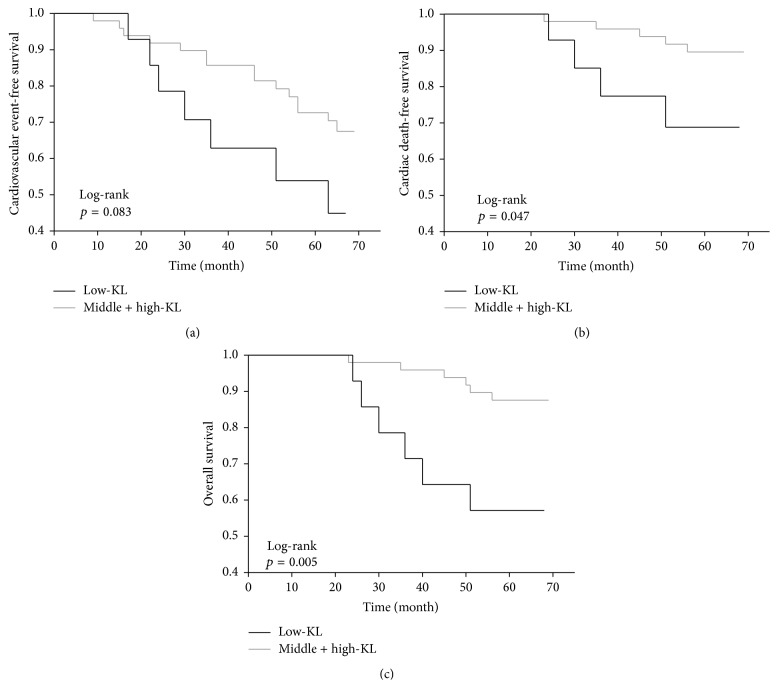
Cardiovascular event-free survival (a), cardiovascular mortality (b), and cumulative survival (c) by the 25th percentile of serum soluble Klotho. Note that the patients categorized into the low-KL (<309 pg/mL) group had poorer cardiovascular (*p* = 0.047) and cumulative survival (*p* = 0.005) rates.

**Figure 3:**
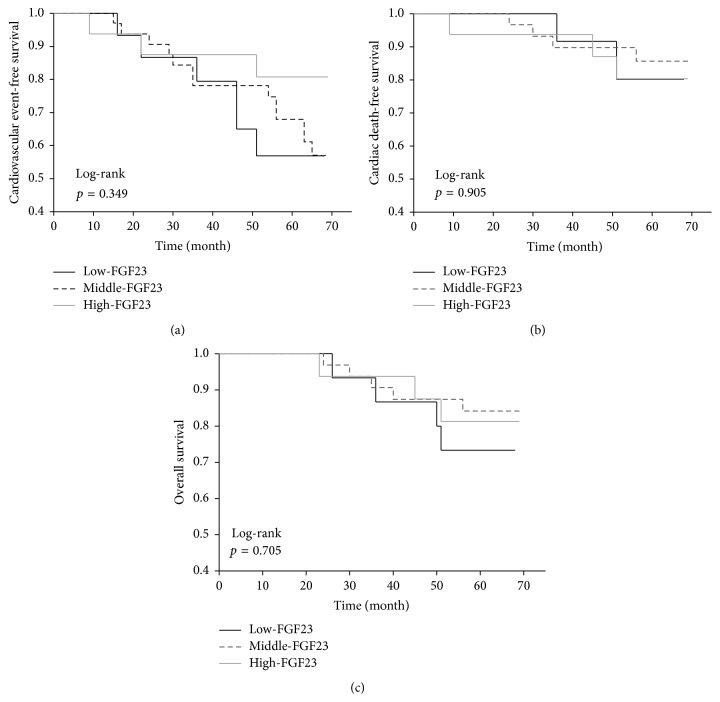
Significance of the serum FGF23 levels with respect to cardiovascular event-free survival (a), cardiovascular mortality (b), and cumulative survival (c). The patients were categorized into low-FGF23 (<1184 pg/mL), middle-FGF23 (1184 to <15134 pg/mL), and high-FGF23 (≥15134 pg/mL) groups. The Kaplan-Meier analysis with the log-rank test failed to demonstrate any significant differences between the groups.

Twelve patients (19%) died within the median follow-up period of 65 months (IR 63–67). Four patients in the low-KL group, three in the middle-KL group, and two in the high-KL group died of cardiovascular events, while one patient in the low-KL group died of infectious disease. Only two patients in the low-KL group died of carcinoma, versus one patient in the middle-KL group. The serum Klotho levels of the nonsurvivors at study entry were numerically lower (median 319 pg/mL, IR 254–426) than those of the survivors (median 380 pg/mL, IR 337–449). The Kaplan-Meier analysis also showed that the cardiovascular event-free survival and mortality rates did not differ significantly between the three groups, whereas the cumulative survival rate was significantly lower in the low-KL group (Figure 1). The cardiovascular and all-cause mortality rates were significantly higher in the low-KL group than in the middle-KL and high-KL groups, despite the absence of statistical differences in the cardiovascular event-free survival rates between these two groups (Figure 2). The univariate Cox regression analyses revealed several factors to be associated with cardiovascular events, cardiovascular mortality, and all-cause mortality, while only age and a low serum sKlotho level (<309 pg/mL) were predictive for all-cause mortality (Table 2). After adjusting for age, a low serum sKlotho level remained an independent predictor of all-cause mortality (HR 4.17, 95% CI 1.29–13.48, *p* = 0.018). This trend remained significant even after adjusting for age, gender, diabetes mellitus, sPi, sAlb, Hb, 1,25(OH)_2_D, and FGF23 (HR 6.33, 95% CI 1.70–25.44; *p* = 0.0065). The cardiovascular event-free survival, cardiovascular mortality, and all-cause mortality rates did not differ between the low-FGF23 (FGF23 < 1184 pg/mL), middle-FGF23 (1184 ≤ FGF23 < 15134 pg/mL), and high-FGF23 (FGF23 ≥ 15134 pg/mL) groups (Figure 3).

## 4. Discussion 

In the current study, we demonstrated that chronic HD subjects have worse cardiovascular and all-cause mortality rates with low serum sKlotho levels than those without low serum sKlotho levels. Moreover, our results showed that a reduced level of serum sKlotho is associated with all-cause mortality, even after adjusting for confounding variables, implying that the circulating sKlotho is a potential predictive indicator of overall mortality among patients with end-stage renal disease (ESRD). Previous findings have demonstrated that low serum sKlotho is associated with adverse kidney disease outcomes and/or arterial stiffness in some subsets of CKD patients and is related to all-cause mortality in older community-dwelling adults [14, 15, 25, 26]. However, one may argue against our hypothesis since the intrinsic role of serum sKlotho levels in predicting adverse outcomes and the disturbed mineral metabolism noted in subjects with various degrees of CKD has been demonstrated to be marginal [16, 27–29]. Seiler et al. reported that sKlotho in patients with CKD stages 2–4 was not significantly related to cardiovascular events and mortality [29, 30]. A recent study by Buiten et al. reported that serum sKlotho in dialysis patients (CKD 5D) was not independently associated with the presence of cardiovascular disease, although patients with low serum sKlotho demonstrate a high rate of coronary artery disease and left ventricular dysfunction [31]. However, this study differs from ours in several respects: it is a cross-sectional study; it includes peritoneal dialysis patients (33% of entries); it evaluated a shorter dialysis vintage (mean 2.3 years); and it measured a relatively high residual renal function (mean creatinine 7.5 mg/dL). Another study by Nowak et al. also showed that sKlotho is not associated with mortality in HD patients [16]. However, in this study, the rate of total cardiovascular complications at intake was higher at 80.0% than in our study at 12.7% [16]. If a low sKlotho predicts cardiovascular events and mortality, a high rate of cardiovascular complications at enrollment may mask its impact. In addition, the follow-up period of the study was shorter at about 2.5 years than ours at more than 5 years [16]. Importantly, we followed chronic HD patients (mean dialysis vintage of 6.7 years) with lower residual renal function (mean creatinine 10.6 mg/dL) for a longer time, which might lead to results at variance with previous studies [16, 31]. Our results also imply the presence of a latent threshold value, which is under the median (25th percentile), for the serum sKlotho level associated with a higher risk of mortality. We believe that our study provides a novel finding to consider the role of low serum sKlotho for a long-term adverse outcome in HD patients.

Information regarding the kinetics of circulating sKlotho has been poorly understood [5, 27, 29]. Our previous findings demonstrating approximately 30% to 50% decreases in the serum sKlotho levels after nephrectomy in living donors indicate that the kidney is a major source of circulating sKlotho in humans without deteriorated renal function [7]. Similarly, recent animal studies have shown that the kidney is the principal contributor of sKlotho [32, 33]. Moreover, recent demonstrations of Klotho expression in vascular tissue highlight the vascular territory as another source of sKlotho [34]. The magnitude of functioning nephrons may also be linked to the level of Klotho in the circulation [5, 35]. It has been documented that the expression of Klotho is decreased in various tissues in patients with CKD [4, 36, 37], and reduced Klotho expression within the parathyroid tissue has been demonstrated in advanced CKD patients [36]. The vascular smooth muscle also appears to be Klotho-deficient in patients with CKD [4]. Therefore, the fact that serum sKlotho levels are lower in chronic HD patients than in healthy human subjects may not be surprising [16, 38]. Indeed, an association between lower serum sKlotho and more advanced CKD has been shown in several cross-sectional observations [5, 25, 26, 35, 39]. However, previous findings demonstrating that the renal Klotho expression levels in patients with CKD are as low as 5% of those observed in persons with normal kidney function [37] led us to consider that serum sKlotho levels appear to be disproportionately high in chronic HD patients, including our study population possibly due to extrarenal production or impaired metabolic clearance of sKlotho, or some combination of the two. Similar disproportionately high levels of sKlotho were found in previous studies of dialysis patients [16, 27, 31, 40]. In this context, the level of sKlotho in the circulation may not necessarily mirror the Klotho expression observed at the tissue level, particularly in advanced CKD patients.

The soluble form of Klotho may target multiple remote tissues and organs, comprising a wide range of biological activities, including antiaging properties [41, 42]. It has been demonstrated that recombinant Klotho proteins of the full length of the extracellular domain inhibit insulin-like growth factor 1 (IGF-1) signal transduction, thus increasing longevity in mice [8]. Moreover, the blockade of IGF-1 signaling induced by the released form of Klotho has been shown to increase resistance to oxidative stress, thereby improving survival [43]. Several lines of evidence have focused on the antioxidative actions of Klotho as a potential therapeutic strategy for treating various kinds of tissue and vascular injuries [44], and a link between the life-extending and the oxidative stress-reducing effects of Klotho has been recognized [45]. In addition, Klotho directly and/or indirectly promotes endothelial nitric oxide synthesis, thus protecting the cardiovascular system [46, 47]. Although whether circulating sKlotho works as a biologically active molecule in chronic HD patients remains to be delineated, the present findings led us to consider that some of the specific roles of Klotho may also apply in the chronic HD patients included in the current observation. A recent noteworthy study showed that a decreased level of circulating sKlotho in CKD mice is an important cause of uremic cardiomyopathy independent of FGF23 and phosphate and that intravenous delivery of a transgene encoding sKlotho ameliorated the cardiomyopathy [18]. This study implies the critical role of low sKlotho for adverse cardiovascular outcome in ESRD patients. Another recent study demonstrated that restoring a diminished Klotho expression confers human aortic smooth muscle cells with an anticalcific nature by improving FGF23-mediated signaling [34]. Moreover, it is interesting to note that vascular Klotho deficiency contributes to vascular calcification, which has been shown to be associated with cardiovascular mortality in patients with CKD [4]. Nevertheless, further studies are required to evaluate the impact of Klotho on the overall survival of chronic HD patients taking into consideration that ESRD patients have higher mortality rates than the general population and cardiovascular disease has received attention as a leading cause of death in patients under chronic HD treatment [48, 49].

Several studies in CKD patients have shown that circulating FGF23, a hormone involved in phosphorous and vitamin D homeostasis, predicts adverse outcome [29, 30, 50, 51]. Among patients with CKD stages 2–4, circulating FGF-23 is significantly associated with cardiovascular outcomes [29, 30, 50]. Gutiérrez et al. showed that rising FGF23 levels in patients beginning HD (CKD 5D) are independently associated with high mortality [51]. Furthermore, a study with animal data showed that FGF23 induces left ventricular hypertrophy via FGF receptor-dependent activation [52]. Our data, however, showed that serum FGF23 did not predict cardiovascular disease and mortality. Unlike previous reports, we focused on long-term HD patients (mean HD period 6.7 ± 5.4 years) who have little urine volume. FGF23 increases urinary phosphorus excretion and inhibits activation of vitamin D in the renal proximal tubule [53], but these physiological effects of FGF23 on renal function may not be exerted in ESRD patients with little urine volume. In other words, the function and regulation of FGF23 in patients with deteriorated renal function are unknown. We speculate that the absence of correlation between FGF23 and clinical outcomes in our study may be a unique feature in patients with long-term maintained HD.

## 5. Conclusions

In this study, we demonstrate for the first time that chronic hemodialysis subjects with low serum soluble Klotho levels have worse cardiovascular and all-cause mortality rates than those without low soluble Klotho levels. Moreover, we found that reduced serum soluble Klotho is associated with all-cause mortality, even after adjusting for confounding variables, implying that the level of serum soluble Klotho plays a role as a predictive indicator of overall mortality in patients with ESRD.

## Figures and Tables

**Table 1:** Demographic and clinical characteristics of patients according to study group.

Characteristics	Total	Low-Klotho [Klotho < 309 pg/mL]	Middle-Klotho [309 ≤ Klotho < 449 pg/mL]	High-Klotho [449 pg/mL ≤ Klotho]	*p* value
Number	63	14	35	14	
Age (year)	64.2 ± 13.0	66.1 ± 11.9	63.8 ± 13.7	63.0 ± 13.0	0.80
Male gender (%)	50.8	42.9	54.3	50.0	0.77
BMI (kg/m^2^)	21.3 ± 3.2	20.3 ± 3.0	21.8 ± 3.2	20.9 ± 3.2	0.29
Diabetes mellitus (%)	33.3	42.9	37.1	14.3	0.18
Duration of dialysis (year)	6.7 ± 5.4	6.2 ± 4.0	6.5 ± 5.2	7.6 ± 7.1	0.77
History of cardiovascular disease (%)	12.7	7.1	11.4	21.4	0.52
Smoking (%)	62.9	64.3	58.8	71.4	0.70
Serum creatinine	10.6 ± 2.8	9.9 ± 0.9	10.7 ± 2.8	10.9 ± 0.8	0.63
*Kt*/*V* (per week)	1.36 ± 0.28	1.47 ± 23	1.33 ± 0.28	1.31 ± 0.29	0.23
nPCR (g/kg/day)	0.95 ± 0.18	0.97 ± 13	0.94 ± 0.20	0.94 ± 0.17	0.91
Cardiothoracic ratio (%)	50.0 ± 5.3	50.8 ± 6.5	49.5 ± 4.8	50.1 ± 5.6	0.79
Systolic blood pressure (mmHg)	154.1 ± 20.0	150.9 ± 20.4	156.3 ± 21.7	151.9 ± 15.5	0.63
Hemoglobin (g/dL)	9.7 ± 1.3	9.5 ± 1.2	9.8 ± 1.4	9.5 ± 1.2	0.64
Serum albumin (g/dL)	3.74 ± 0.36	3.61 ± 0.39	3.76 ± 0.34	3.84 ± 0.39	0.23
Total cholesterol (mg/dL)	158 ± 1.3	153 ± 32	165 ± 36	143 ± 0.35	0.12
Serum calcium (mg/dL)	8.9 ± 0.2	8.6 ± 1.3	9.0 ± 1.2	8.9 ± 1.3	0.60
Serum phosphorus (mg/dL)	5.0 ± 0.2	4.6 ± 1.2	4.9 ± 1.4	5.6 ± 1.0	0.11
Intact PTH (pg/mL)	99 (45–250)	96 (47–210)	94 (42–250)	185 (79–298)	0.35
Klotho (pg/mL)	371 (309–449)	245 (220–283)	371 (341–401)	550 (468–692)	<0.0001
Serum FGF23 (pg/mL)	4511 (1184–15134)	2115 (609–4422)	4844 (779–17492)	9036 (2815–21678)	0.02
1,25(OH)_2_D (pg/mL)	8.6 ± 4.4	7.7 ± 3.6	8.4 ± 4.5	9.7 ± 4.9	0.46
Vitamin D sterols (%)	46.0	50.0	48.6	35.7	0.67
Phosphate binders (%)	93.7	100	88.6	100	0.09
ESA (%)	91.9	100	88.2	92.9	0.23
ARBs/ACEIs (%)	47.6	21.4	48.6	71.4	0.03
CCBs (%)	50.8	42.9	51.4	57.1	0.75
Statin (%)	9.5	0	11.4	14.3	0.20

Variables are presented as numbers of patients (percentage), as mean ± SD, or as median (interquartile range), as appropriate. Klotho: serum soluble Klotho; BMI: body mass index; nPCR: normalized protein catabolism rate; PTH: parathyroid hormone; FGF23: fibroblast growth factor 23; ESA: erythropoiesis stimulating agent; ARBs: angiotensin receptor blockers; ACEIs: angiotensin converting enzyme inhibitors; CCBs: calcium channel blockers.

**Table 2:** Univariate Cox regression analyses of cardiovascular events, cardiovascular mortality, and all-cause mortality.

Parameter	Cardiovascular events		Cardiovascular mortality		All-cause mortality	
	Hazard ratio (95% CI)	*p* value	Hazard ratio (95% CI)	*p* value	Hazard ratio (95% CI)	*p* value
Age (year)	1.06 (1.02–1.11)	0.004	1.13 (1.04–1.26)	0.002	1.09 (1.02–1.18)	0.005
Male gender	1.52 (0.65–3.68)	0.33	2.02 (0.53–9.57)	0.31	2.01 (0.63–7.53)	0.24
BMI (kg/m^2^)	1.11 (0.98–1.25)	0.10	0.99 (0.79–1.20)	0.93	1.00 (0.83–1.18)	0.97
Diabetes mellitus	1.42 (0.59–3.30)	0.42	1.70 (0.42–6.41)	0.43	2.12 (0.66–6.77)	0.20
Duration of dialysis (year)	0.95 (0.85–1.03)	0.23	0.94 (0.77–1.07)	0.40	0.94 (0.81–1.06)	0.36
Serum creatinine	0.97 (0.83–1.12)	0.64	0.95 (0.74–1.19)	0.65	0.90 (0.72–1.10)	0.30
*Kt*/*V* (per/week)	0.23 (0.06–1.00)	0.05	0.69 (0.08–7.40)	0.75	0.80 (0.12–6.29)	0.83
nPCR (g/kg/day)	0.05 (0.005–0.61)	0.02	1.72 (0.05–62.78)	0.77	1.54 (0.06–39.72)	0.79
Cardiothoracic ratio (%)	1.09 (1.01–1.18)	0.02	1.15 (1.02–1.29)	0.02	1.09 (0.98–1.21)	0.10
Systolic blood pressure (mmHg)	1.00 (0.98–1.02)	0.93	1.00 (0.97–1.03)	0.95	1.00 (0.98–1.03)	0.75
Hemoglobin (g/dL)	0.90 (0.65–1.26)	0.54	0.35 (0.01–11.63)	0.55	0.95 (0.62–1.45)	0.80
Serum albumin (g/dL)	0.55 (0.18–1.68)	0.29	1.11 (0.19–7.42)	0.91	1.14 (0.25–5.81)	0.87
Total cholesterol (mg/dL)	0.99 (0.97–1.00)	0.06	0.98 (0.96–1.00)	0.10	0.99 (0.98–1.01)	0.47
Serum calcium (mg/dL)	0.83 (0.59–1.18)	0.30	0.72 (0.40–1.23)	0.23	0.85 (0.52–1.34)	0.48
Serum phosphorus (mg/dL)	0.83 (0.61–1.14)	0.25	0.94 (0.57–1.54)	0.80	0.82 (0.54–1.26)	0.36
Intact PTH (pg/mL)	1.00 (0.99–1.00)	0.91	1.00 (0.998–1.004)	0.35	1.00 (0.997–1.003)	0.79
Klotho (Low)	2.17 (0.83–5.18)	0.11	3.50 (0.86–13.26)	0.08	4.38 (1.37–14.04)	0.014
Serum FGF23 (High)	1.33 (0.57–3.22)	0.51	1.95 (0.52–9.25)	0.33	0.98 (0.31–3.15)	0.98
1,25(OH)_2_D (pg/mL)	1.00 (0.90–1.09)	0.96	1.07 (0.92–1.21)	0.34	1.04 (0.90–1.17)	0.96
Vitamin D sterols (No)	0.65 (0.27–1.50)	0.31	0.41 (0.09–1.56)	0.19	0.59 (0.17–1.84)	0.36
Phosphate binders (No)	1.48 (0.24–5.09)	0.61	2.01 (3.14–3.14)	0.25	2.01 (2.29–2.29)	0.19
ESA (No)	0.94 (0.27–1.50)	0.93	1.94 (2.42–2.42)	0.19	1.94 (1.76–1.76)	0.13
ARBs/ACEIs (No)	1.57 (0.68–3.82)	0.29	3.66 (0.88–24.60)	0.08	3.15 (0.94–14.22)	0.06

BMI: body mass index; nPCR: normalized protein catabolism rate; PTH: parathyroid hormone; Klotho: serum soluble Klotho; FGF23: fibroblast growth factor 23; ESA: erythropoiesis stimulating agent; ARBs: angiotensin receptor blockers; ACEIs: angiotensin converting enzyme inhibitors; CCBs: calcium channel blockers.

## References

[1] Hu M. C., Shiizaki K., Kuro-O M., Moe O. W. (2013). Fibroblast growth factor 23 and Klotho: physiology and pathophysiology of an endocrine network of mineral metabolism. *Annual Review of Physiology*.

[2] Kuro-o M., Matsumura Y., Aizawa H. (1997). Mutation of the mouse klotho gene leads to a syndrome resembling ageing. *Nature*.

[3] Imura A., Iwano A., Tohyama O. (2004). Secreted Klotho protein in sera and CSF: implication for post-translational cleavage in release of Klotho protein from cell membrane. *FEBS Letters*.

[4] Hu M. C., Shi M., Zhang J. (2011). Klotho deficiency causes vascular calcification in chronic kidney disease. *Journal of the American Society of Nephrology*.

[5] Akimoto T., Yoshizawa H., Watanabe Y. (2012). Characteristics of urinary and serum soluble Klotho protein in patients with different degrees of chronic kidney disease. *BMC Nephrology*.

[6] Akimoto T., Shiizaki K., Sugase T. (2012). The relationship between the soluble Klotho protein and the residual renal function among peritoneal dialysis patients. *Clinical and Experimental Nephrology*.

[7] Akimoto T., Kimura T., Watanabe Y. (2013). The impact of nephrectomy and renal transplantation on serum levels of soluble Klotho protein. *Transplantation Proceedings*.

[8] Kurosu H., Yamamoto M., Clark J. D. (2005). Suppression of aging in mice by the hormone Klotho. *Science*.

[9] Arking D. E., Becker D. M., Yanek L. R. (2003). KLOTHO allele status and the risk of early-onset occult coronary artery disease. *The American Journal of Human Genetics*.

[10] Arking D. E., Atzmon G., Arking A., Barzilai N., Dietz H. C. (2005). Association between a functional variant of the KLOTHO gene and high-density lipoprotein cholesterol, blood pressure, stroke, and longevity. *Circulation Research*.

[11] Yamada Y., Ando F., Niino N., Shimokata H. (2005). Association of polymorphisms of the androgen receptor and klotho genes with bone mineral density in Japanese women. *Journal of Molecular Medicine*.

[12] Friedman D. J., Afkarian M., Tamez H. (2009). Klotho variants and chronic hemodialysis mortality. *Journal of Bone and Mineral Research*.

[13] Ko G. J., Lee Y. M., Lee E. A. (2013). The association of Klotho gene polymorphism with the mortality of patients on maintenance dialysis. *Clinical Nephrology*.

[14] Semba R. D., Cappola A. R., Sun K. (2011). Plasma klotho and cardiovascular disease in adults. *Journal of the American Geriatrics Society*.

[15] Semba R. D., Cappola A. R., Sun K. (2011). Plasma klotho and mortality risk in older community-dwelling adults. *Journals of Gerontology—Series A: Biological Sciences and Medical Sciences*.

[16] Nowak A., Friedrich B., Artunc F. (2014). Prognostic value and link to atrial fibrillation of soluble klotho and FGF23 in hemodialysis patients. *PLoS ONE*.

[17] Hu M. C., Shi M., Cho H. J. (2015). Klotho and phosphate are modulators of pathologic uremic cardiac remodeling. *Journal of the American Society of Nephrology*.

[18] Xie J., Yoon J., An S. W., Kuro O. M., Huang C. L. (2015). Soluble klotho protects against uremic cardiomyopathy independently of fibroblast growth factor 23 and phosphate. *Journal of the American Society of Nephrology*.

[19] Masuda T., Murata M., Honma S. (2011). Sleep-disordered breathing predicts cardiovascular events and mortality in hemodialysis patients. *Nephrology Dialysis Transplantation*.

[20] Shinzato T., Nakai S., Fujita Y. (1994). Determination of Kt/V and protein catabolic rate using pre- and postdialysis blood urea nitrogen concentrations. *Nephron*.

[21] Thygesen K., Alpert J. S., Jaffe A. S. (2012). Third universal definition of myocardial infarction. *Journal of the American College of Cardiology*.

[22] Mcmurray J. J. V., Adamopoulos S., Anker S. D. (2012). ESC guidelines for the diagnosis and treatment of acute and chronic heart failure 2012: the Task Force for the Diagnosis and Treatment of Acute and Chronic Heart Failure 2012 of the European Society of Cardiology. Developed in collaboration with the Heart Failure Association (HFA) of the ESC. *European Journal of Heart Failure*.

[23] Easton J. D., Saver J. L., Albers G. W. (2009). Definition and evaluation of transient ischemic attack: a scientific statement for healthcare professionals from the American heart association/American stroke association stroke council; council on cardiovascular surgery and anesthesia; council on cardiovascular radiology and intervention; council on cardiovascular nursing; and the interdisciplinary council on peripheral vascular disease. *Stroke*.

[24] Sacco R. L., Kasner S. E., Broderick J. P. (2013). An updated definition of stroke for the 21st century: a statement for healthcare professionals from the American heart association/American stroke association. *Stroke*.

[25] Kitagawa M., Sugiyama H., Morinaga H. (2013). A decreased level of serum soluble Klotho is an independent biomarker associated with arterial stiffness in patients with chronic kidney disease. *PLoS ONE*.

[26] Kim H. R., Nam B. Y., Kim D. W. (2013). Circulating alpha-klotho levels in CKD and relationship to progression. *American Journal of Kidney Diseases*.

[27] Komaba H., Koizumi M., Tanaka H. (2012). Effects of cinacalcet treatment on serum soluble Klotho levels in haemodialysis patients with secondary hyperparathyroidism. *Nephrology, Dialysis, Transplantation*.

[28] Fliser D., Seiler S., Heine G. H., Ketteler M. (2012). Measurement of serum soluble Klotho levels in CKD 5D patients: useful tool or dispensable biomarker?. *Nephrology, Dialysis, Transplantation*.

[29] Seiler S., Wen M., Roth H. J. (2013). Plasma Klotho is not related to kidney function and does not predict adverse outcome in patients with chronic kidney disease. *Kidney International*.

[30] Seiler S., Rogacev K. S., Roth H. J. (2014). Associations of FGF-23 and sKlotho with cardiovascular outcomes among patients with CKD stages 2–4. *Clinical Journal of the American Society of Nephrology*.

[31] Buiten M. S., de Bie M. K., Bouma-de Krijger A. (2014). Soluble Klotho is not independently associated with cardiovascular disease in a population of dialysis patients. *BMC Nephrology*.

[32] Hu M. C., Shi M., Zhang J. (2015). Renal production, uptake, and handling of circulating *α*Klotho. *Journal of the American Society of Nephrology*.

[33] Lindberg K., Amin R., Moe O. W. (2014). The kidney is the principal organ mediating klotho effects. *Journal of the American Society of Nephrology*.

[34] Lim K., Lu T.-S., Molostvov G. (2012). Vascular klotho deficiency potentiates the development of human artery calcification and mediates resistance to fibroblast growth factor 23. *Circulation*.

[35] Rotondi S., Pasquali M., Tartaglione L. (2015). Soluble *α*-Klotho serum levels in chronic kidney disease. *International Journal of Endocrinology*.

[36] Komaba H., Goto S., Fujii H. (2010). Depressed expression of Klotho and FGF receptor 1 in hyperplastic parathyroid glands from uremic patients. *Kidney International*.

[37] Koh N., Fujimori T., Nishiguchi S. (2001). Severely reduced production of klotho in human chronic renal failure kidney. *Biochemical and Biophysical Research Communications*.

[38] Yokoyama K., Imura A., Ohkido I. (2012). Serum soluble *α*-klotho in hemodialysis patients. *Clinical Nephrology*.

[39] Shimamura Y., Hamada K., Inoue K. (2012). Serum levels of soluble secreted a-Klotho are decreased in the early stages of chronic kidney disease, making it a probable novel biomarker for early diagnosis. *Clinical and Experimental Nephrology*.

[40] Takahashi H., Komaba H., Takahashi Y. (2014). Impact of parathyroidectomy on serum FGF23 and soluble klotho in hemodialysis patients with severe secondary hyperparathyroidism. *Journal of Clinical Endocrinology and Metabolism*.

[41] Drüeke T. B., Massy Z. A. (2013). Circulating Klotho levels: clinical relevance and relationship with tissue Klotho expression. *Kidney International*.

[42] Hu M. C., Kuro-o M., Moe O. W. (2012). Secreted klotho and chronic kidney disease. *Advances in Experimental Medicine and Biology*.

[43] Yamamoto M., Clark J. D., Pastor J. V. (2005). Regulation of oxidative stress by the anti-aging hormone klotho. *Journal of Biological Chemistry*.

[44] Akimoto T., Morishita Y., Ito C. (2014). Febuxostat for hyperuricemia in patients with advanced chronic kidney disease. *Drug Target Insights*.

[45] Finkel T., Holbrook N. J. (2000). Oxidants, oxidative stress and the biology of ageing. *Nature*.

[46] Saito Y., Yamagishi T., Nakamura T. (1998). Klotho protein protects against endothelial dysfunction. *Biochemical and Biophysical Research Communications*.

[47] Saito Y., Nakamura T., Ohyama Y. (2000). In vivo klotho gene delivery protects against endothelial dysfunction in multiple risk factor syndrome. *Biochemical and Biophysical Research Communications*.

[48] Goldsmith D. (2008). Negative outcome studies in end-stage renal disease. *Blood Purification*.

[49] Neovius M., Jacobson S. H., Eriksson J. K., Elinder C.-G., Hylander B. (2014). Mortality in chronic kidney disease and renal replacement therapy: a population-based cohort study. *BMJ Open*.

[50] Scialla J. J., Xie H., Rahman M. (2014). Fibroblast growth factor-23 and cardiovascular events in CKD. *Journal of the American Society of Nephrology*.

[51] Gutiérrez O. M., Mannstadt M., Isakova T. (2008). Fibroblast growth factor 23 and mortality among patients undergoing hemodialysis. *The New England Journal of Medicine*.

[52] Faul C., Amaral A. P., Oskouei B. (2011). FGF23 induces left ventricular hypertrophy. *Journal of Clinical Investigation*.

[53] Ix J. H., Katz R., Kestenbaum B. R. (2012). Fibroblast growth factor-23 and death, heart failure, and cardiovascular events in community-living individuals: CHS (Cardiovascular Health Study). *Journal of the American College of Cardiology*.

